# Voluntary Chaplains

**Published:** 1888-09-29

**Authors:** 


### The Hospital

A Weekly Institutional Journal of

**Science, Medicine, Nursing, and Philanthropy.**

*For Hospitals, Asylums, Medical Practitioners, Students, Nurses, Families, Parishes, Congregations, and the Charitable Public.*

Vol. IV. No. 105.] [September 29, 1888.

### VOLUNTARY CHAPLAINS.

The Rev. C. Watson, Chaplain to the York County Hospital, has resigned his position, in consequence of increasing age and infirmity. Following on his resignation, an advertisement was inserted in the local newspapers for a successor. In response to the advertisement only one candidate, the Rev. S. Commeline, B.A., made application. Mr. Commeline is one of the vicars-choral of the minster, and, it may be presumed, is in every way suitable for the vacant post. When, however, the candidate's name was put to the vote, the "ayes" were in a minority, and consequently the York County Hospital is now without a chaplain. We gather from a leader in a local newspaper that there is a strong feeling on the part of many of the supporters of the institution that the days of paid chaplains in voluntary hospitals are numbered, and that in future it may be both hoped and expected that chaplains will be, like visiting physicians and surgeons, purely honorary officers.

A question is thus raised of great interest to the whole community, as well as to the supporters of the York County Hospital. It seems certain that in future endowed hospitals will be relatively few, and that voluntary hospitals will practically cover the whole field of medical charity. It appears to be equally certain that the support of such institutions will not be less, but more difficult as time advances. There are many reasons for this. The resources of individuals tend to diminish as they are shared by an increasing population. Living becomes more expensive as the standard of comfort and convenience is raised. Hospitals themselves grow more costly, and the demands on their resources more urgent and excessive. All these things considered, it is evident we have to look forward to a time of increasing necessities and diminished powers of meeting them. The *res angusta* of hospitals is a fact which, now urgent, tends always to become more and more intense.

Men of foresight, who desire to see the toiling millions properly cared for in times of sickness, look ahead with much perturbation of spirit. It is evident to them, however blind others may be, that unless something can be done either to increase resources or to diminish expenditure or to lessen claims, there will be a deadlock of the most absolute and painful character. However callous some races and some countries may be to the sufferings of the toiling and helpless poor, with us it is as necessary that the sick should have skilled aid as that the hungry should have daily bread. Unless the character of the people of Great Britain should undergo a complete retrogression, the public conscience and sentiment will demand that hospitals be maintained at least in their present, if not in a more advanced, state of efficiency. We have shown however that resources are not likely to keep pace with demands, but that, on the contrary, whilst claims extend in an ever-increasing ratio, the means of meeting them show undoubted indications of shrinkage on all sides. There is, therefore, only one other direction in which practical men may look with the hope of an adjustment which shall restore the balance between distress and relief. The outlook must undoubtedly be in the direction of economy. Not only must superfluities be absolutely cut off, but the less urgent necessities must give place to the more urgent until hospital expenditure is brought to the irreducible minimum, and nothing is given in medical charity except that which is indispensable to the medical treatment of the sick.

We are thus brought face to face with the question of what are the absolute indispensables for the proper treatment of the sick? It is an interrogation which covers a wide area, and which cannot be profitably discussed in all its bearings in a single article. For the present the inquiry will be restricted to the case of paid chaplains. Are paid chaplains indispensable to the proper treatment of the sick in hospitals? This is not the same as asking if religious ministrations are desirable or essential. If it were, there could be but one answer, and that of an affirmative kind. It is granted at once that the sick who are treated at hospitals ought to have the opportunity of attending religious services at regular periods, and that at special times they should be able to secure such additional spiritual aid and comfort as they may wish for. If man is a religious being always and everywhere, most of all does he incline to the consolations of religion when he is sick or dying. That will be freely and unhesitatingly admitted by all but extreme and unreasoning partisans. But, granted that hospital patients must have spiritual counsel, is there no other way of securing it for them except by means of paid chaplains? Undoubtedly there is! In the case of physicians and surgeons there are always found far more than sufficient for the vacant posts of honorary officers. Will it be contended that the same would not hold good of clergymen and ministers of religion? It may be said that the doctor has something to gain by his hospital appointment, and that therefore there is no lack of suitable applicants. And has the clergyman nothing to gain? Has he so little faith in the religion he teaches that its rewards are too shadowy for his appreciation? We refuse to believe it. All experience is against it. There need be no manner of doubt that hospitals both in large and small towns would be able to secure much more voluntary spiritual aid than they would require if the office of paid chaplain were abolished forthwith. At York the suggestion is made, not that one clergyman shall do all the duties of voluntary chaplain, but that ten, fifteen, twenty, or more shall divide the duties among them, each of them taking the regular work and holding himself in readiness for extraordinary calls for a definite period. This suggestion seems entirely possible, and the York Hospital will render a great service to the public if it will give a decided lead in the new direction.

Two important results would probably immediately follow the abolition of paid chaplaincies at voluntary hospitals. In the first place, there would be the undoubted saving of the chaplain's salary. Such a saving would, in many instances, be sufficient to turn the annual balance in the hospital accounts from the debit to the credit side. That of itself would be a grateful relief to the voluntary managers as well as the necessary paid officials, changing the annual meeting into a day of mutual congratulation from one of general anxiety and gloom. But the second would be a far more important and fruitful result than one of mere money-saving. It would satisfy the subscribers that none of their money was being spent on things that could be otherwise provided. It would let them see that they are not the only peeople who can deny themselves for the sake of benefiting the sick poor. It would show the members of committee, many of whom devote not only a large proportion of their leisure, but a considerable share of their means to the hospital, that they are not the sole volunteers, but that others can give of what they have, even if what they give is not in money or business capacity. It would, we think, be of service to the clergy themselves. They would experience a sense of satisfaction and pleasure in voluntarily ministering to the poor and sick who have no claims upon them of parish or congregation which would more than repay them for the time and labour spent in such a service. Probably, too, the patients would profit decidedly by the change, because they would feel that the minister of religion who sought to cheer their bed of sickness, was not there because it was his prescribed duty which he performed for the wages of the hireling, but because he felt for them the heartfelt sympathy of a brother man, and the still tenderer solicitude of one who had a divine message of comfort to deliver to a soul in suffering and sorrow. We do not counsel any sudden changes; still less do we desire any man to be deprived of emoluments to which he has been accustomed, and which may be indispensable for his needs. What we wish is to call attention to the question, and to suggest that, as hospital chaplaincies become vacant, the experiment shall be made of trying to supply the spiritual needs of the patients by generous-hearted, voluntary services. If the York County Hospital will lead the way an example will be set which, it may be hoped, will soon be generally followed.

